# Effect and Safety of Therapeutic Regimens for Patients With Germline BRCA Mutation-Associated Breast Cancer: A Network Meta-Analysis

**DOI:** 10.3389/fonc.2021.718761

**Published:** 2021-08-20

**Authors:** Ying Jiang, Xiang-Yu Meng, Ning-Ning Deng, Chen Meng, Lu-Hui Li, Zi-Kang He, Xing-Yun Wang, Zhe-Yao Song, Rong-Jun Cui

**Affiliations:** ^1^Department of Biochemistry and Molecular Biology, Mudanjiang Medical University, Mudanjiang, China; ^2^Department of Dermatological, Hongqi Hospital Affiliated to Mudanjiang Medical University, Mudanjiang, China; ^3^Department of Nursing Youth League Committee, Mudanjiang Medical University, Mudanjiang, China

**Keywords:** BRCA mutation, chemotherapy, targeted therapy, meta-analysis, breast cancer

## Abstract

**Purpose:**

Breast cancer type 1 susceptibility (BRCA) mutations not only increase breast cancer (BC) risk but also result in poor survival and prognosis for BC patients. This study will analyze the effect and safety of therapeutic regimens for the treatment of BC patients with germline BRCA (gBRCA) mutations by network meta-analysis.

**Methods:**

Public databases were searched from inception to 29 April 2021. Frequentist network meta-analysis was conducted to analyze the benefit of chemotherapy and targeted drug-related strategies.

**Results:**

Seventeen articles were included in the analysis. For progression-free survival (PFS), olaparib (hazard ratio (HR): 0.58; 95% confidence interval (CI): 0.43 – 0.79), platinum (HR: 0.45; 95% CI: 0.22 – 0.89), and talazoparib (HR: 0.54; 95% CI: 0.41 – 0.71) were significantly better than platinum-free chemotherapy (Chemo). The results based on indirect comparisons showed that veliparib (Vel) + platinum + Chemo was also significantly better than Chemo (HR: 0.37; 95% CI: 0.20 – 0.69). For overall survival (OS), olaparib was significantly better than Chemo only in the population who did not receive prior chemotherapy. For pathologic complete response (pCR), bevacizumab+Chemo had a significant advantage over platinum agents (OR: 3.64; 95% CI: 1.07 - 12.39). Olaparib and talazoparib both showed significantly higher objective response rates (ORRs) than Chemo.

**Conclusion:**

The PFS results suggested that olaparib, talazoparib, and Vel+platinum agent+Chemo were ideal regimens for overall, TNBC, and advanced BC patients with gBRCA mutations. Whether PARPis are suitable for patients with gBRCA mutations who have received prior platinum therapy still needs to be clarified.

## Introduction

Breast cancer (BC) is one of the three most common cancers in the world (1) and is also the most common cancer and the leading cause of cancer-related death in women globally (2). Approximately 2.1 million women were newly diagnosed with BC in 2018, with BC accounting for nearly a quarter of female cancer cases worldwide (3).

Breast cancer type 1 susceptibility (BRCA), a tumor suppressor gene, encodes a protein that repairs DNA double-strand breaks by homologous recombination repair (HRR) and can inhibit the occurrence of malignant tumors (4). Germline BRCA1/2 mutation reduces gene expression and has been considered an important risk factor for the onset of BC; in addition, mutant BRCA1/2 allele carriers have a lifetime risk of BC as high as 80%-85% (5). Approximately 75% of BC patients with germline BRCA1 (gBRCA) mutations are classified as having triple-negative breast cancer (TNBC), and the TNBC rate among patients with gBRCA2 mutations is reported to be 20%-25% (6, 7), which often has a high nuclear grade and a larger tumor burden (8). In addition to the risk of BC, BRCA mutations are also considered predive of prognosis, and BC patients with gBRCA mutations have worse survival outcomes than those without gBRCA mutations (9, 10). Therefore, screening for gBRCA mutation carriers may help formulate therapeutic strategies to improve survival outcomes.

Among TNBC patients, the incidence of gBRCA mutation is 11.2% (11). Both gBRCA mutation-associated BC and sporadic TNBC are characterized by abnormal DNA repair ability and extensive genome instability, which support the application of DNA-damaging agents, such as platinum agents (12). Platinum agents can cause DNA strand breaks and lead to cancer cell apoptosis, which makes them more effective in BC cancer cells with DNA repair defects caused by BRCA mutations (13, 14). However, the clinical results are still controversial (15–17).

In addition, for BRCA-mutated human epidermal growth factor receptor 2 (HER2)-negative metastatic or advanced BC, the US Food and Drug Administration (FDA) has approved treatment with the poly (ADP-ribose) polymerase inhibitors (PARPis) talazoparib and olaparib (18). A network meta-analysis showed that these two PARPis have similar effects, safety and acceptability in the treatment of BRCA-mutated HER2-negative metastatic or advanced BC (19).

Because BC patients with gBRCA mutations have defects in homologous recombination repair (HRR), which weakens the ability of cancer cells to repair DNA damage, they are likely to be sensitive to both PARPis and platinum agents (20). In previous meta-analyses, the effectiveness of platinum agents (17, 21) and PARPis (3) was analyzed, but the analysis of combination regimens, patient characteristics, and survival outcomes was inadequate. In particular, no ideal combination regimen containing platinum agents, PARPis and other agents has been identified for BC patients with gBRCA mutations. This study will analyze the effectiveness and safety of various drug regimens for the treatment of BC patients with gBRCA mutations by network meta-analysis.

## Methods

### Search Strategy

The literature search was conducted in PubMed, Embase, Scopus, EBSCOhost and the Cochrane Central Register of Controlled Trials from database inception to 29 April 2021, without language restriction. Keywords included (BRCA*, breast cancer susceptibility gene), (breast, mammary), (malignant, neoplasms, cancer, tumor, carcinoma, adenocarcinoma) and (random*, randomized, randomised). The references of important reviews were also screened to avoid omissions.

### Study Selection and Eligibility Criteria

We included randomized controlled trials (RCTs) of parallel design for BC patients with gBRCA mutations. Eligibility criteria included the following: 1, studies with an RCT design; 2, studies including BC patients with gBRCA mutations or reporting a gBRCA-mutated BC population as a subgroup; 3, studies in which the intervention regimen was chemotherapy or targeted drug-related strategies; and 4, studies reporting survival outcomes in terms of progression-free survival (PFS) or overall survival (OS) based on Cox regression analysis, pathologic complete response (pCR), or objective response rate (ORR). The exclusion criteria included the following: 1, studies without an RCT design; 2, studies that included patients with gBRCA mutations with other types of cancer, such as ovarian cancer, endometrial cancer, or colon cancer; 3, studies that grouped patients by BRCA expression level or methylation instead of by gBRCA mutation; and 4, studies that grouped patients not only by gBRCA mutation but also by other HRR mutations.

### Data Extraction and Quality Assessment

The extracted data included the first author’s name, publication date, research location, study abbreviation, registration ID, patient types, interventions, controls, and follow-up period. Primary outcomes included survival results; secondary outcomes included pCR, ORR, and serious adverse events (SAEs). The Cochrane tool, which includes consideration of random sequence generation, allocation concealment, blinding of investigator and patients, blinding of assessor, missing outcome data, selective reporting and other factors, was used to assess the risk of bias in RCTs (22). The GRADE approach was used to assess the quality of the evidence for direct, indirect and network comparisons. Evidence quality rating factors for comparisons included considerations of risk of bias, inconsistency, indirectness, imprecision and publication bias. If direct and indirect comparison results provided similar results, the higher quality level of the direct and indirect comparisons was used for the network comparison quality level, and if it provided different results, the network comparison quality level was downgraded because of inconsistency. The quality of evidence was divided into high, moderate, low and very low (23).

### Statistical Analysis

Odds ratios (ORs) with corresponding 95% confidence intervals (CIs) were used for dichotomous outcomes, and hazard ratios (HRs) with 95% CIs were used for Cox regression model results of survival analyses. Frequentist network meta-analysis was conducted using random-effects models (24). We assessed agreement between direct and indirect comparisons in every closed loop of evidence using node splitting approaches and for the entire network using a design-by-treatment interaction model. The P-score metric, which measures the extent of certainty that an intervention is better than others, was used to compare the effectiveness and safety of all analyzed therapeutic interventions (25). Study bias was evaluated at the outcome level with Egger’s regression test. Subgroup analysis was performed according to patient mutation type and molecular subtype and receipt of prior chemotherapy or platinum agents. All pairwise and network meta-analyses were performed using RevMan software and R software with the “netmeta” and “meta” packages.

## Results

**Figure 1:**
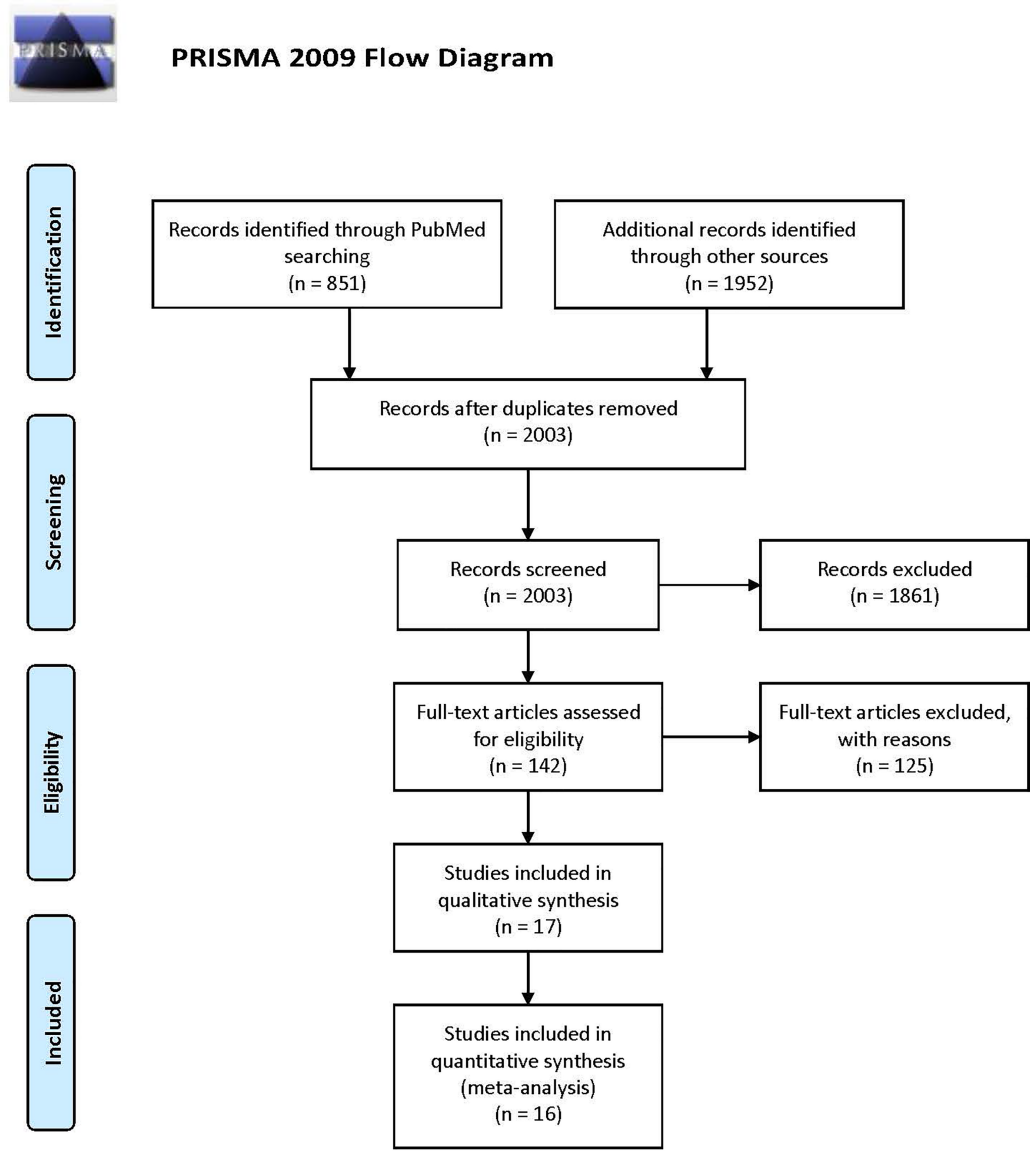
PRISMA flowchart describing the study identification and selection process, which was adopted to document the number of items identified from the databases, the number of duplicates removed, the number of full-text articles screened, and the number of studies included in the final analysis.

After searching, a total of 2,803 items were obtained, and 2,003 items remained after removing duplications. After screening the titles and abstracts, 142 articles were obtained and underwent full-text review. In addition, 125 documents were excluded for the following reasons: duplicate reports (64), studies with no inclusion of a population with gBRCA mutation in the entire population or subgroups (30), studies with no report of the above outcomes or use of the above protocols (16), reviews or comments (9), studies with non-RCT designs (4), studies reporting gBRCA mutation-related BC risk (1), and studies researching populations with mutations in other HRR-related genes (1). Because conference abstracts were included in the search results, many duplicate reports existed. The study abbreviation and registration ID were mainly used for identifying duplicate reports and adopting the latest reported results. Ultimately, 17 articles were included in the analysis (25–41) (**Figure 1** and **Table 1**).

**Table 1:** Characteristics of the included studies.

Study	Location	Study Abbr.	Regist ID	Sample size	Patients’ type	Intervention	Control	Outcomes	Follow-up
LA.Emens 2021 (25)	US	IMpassion130	NCT02425891	89	TNBC	Ate+Chemo	Chemo	PFS;OS	Open
N. Masuda 2021 (26)	Japan	JBCRG-22	UMIN000023162	46	TNBC	Platinum+Chemo	Platinum+Chemo	pCR; ORR; Safety	After neoadjuvant
Ke-Da Yu 2020 (27)	China	PATTERN	NCT01216111	66	TNBC	Platinum+Chemo	Chemo	PFS; OS; Safety	5 Year
Nadine Tung 2020 (28)	USA	INFORM	NCT01670500	118	HER2-negative BC	Chemo	Platinum	pCR; Safety	5 Year
Hope S. Rugo 2020 (29)	USA	EMBRACA	NCT01945775	431	Locally advanced or metastatic BC	Talazoparib	Chemo	PFS;ORR;Safety	Open
Véronique Diéras 2020 (30)	Multicenter	BROCADE3	NCT02163694	509	Advanced HER2-negative BC	Vel+Platinum+Chemo	Platinum+Chemo	PFS;OS; ORR; Safety	Open
Esther Pohl-Rescigno 2020 (31)	German	GeparOcto	NCT02125344	96	TNBC	Chemo	Platinum+Chemo	pCR	Open
PA. Fasching 2020 (32)	German	GeparOLA	NCT02789332	59	HER2-negative BC	Olaparib+Chemo	Platinum+Chemo	pCR	After neoadjuvant
Feng Du 2020 (33)	China	NA	NA	15	TNBC	Platinum+Chemo	Chemo	PFS	5 Year
ME Robson 2019 (34)	Multicenter	OlympiAD	NCT02000622	302	HER2-negative metastatic BC	Olaparib	Chemo	PFS;OS;ORR;Safety	Open
J.Zhang 2018 (35)	China	CBCSG006	NCT01287624	14	Metastatic TNBC	Platinum+Chemo	Chemo	PFS;OS;ORR	54.73 (47.50-60.77) Month
Andrew Tutt 2018 (36)	UK	TNT	NCT00532727	43	Local advanced TNBC	Platinum	Chemo	PFS;OS;ORR;Safety	12 Month
S. Loibl 2018 (37)	Germany	GeparSixto	NCT01426880	54	TNBC	Beva+Chemo	Beva+Platinum+Chemo	PFS;pCR	47.3 (1.7–62.8)Month
S. Loibl 2018 (38)	Multicenter	BrighTNess	NCT02032277	93	TNBC	Vel+Platinum+Chemo	Platinum+Chemo	pCR	4 Year
						Chemo			
HS. Han 2018 (39)	Multicenter	BROCADE	NCT01506609	284	Locally recurrent or metastatic BC	Vel+Platinum+Chemo	Vel+Chemo	PFS;OS;ORR;Safety	Open
						Platinum+Chemo			
PA. Fasching 2018 (40)	German	GeparQuinto	NCT00567554	90	TNBC	Beva+Chemo	Chemo	PFS;pCR	84 Month
PC. Schouten 2015 (41)	Netherlands	NA	NA	41	Stage II-III BC	Platinum+Chemo	Chemo	PFS;OS	Open

Ate, Atezolizumab; BC, Breast cancer; Beva, Bevacizumab; Chemo, Chemotherapy; HER2, human epidermal growth factor receptor 2; NA, not available; ORR, Objective response rate; OS, Overall survival; pCR, pathologic complete response; PFS, Progression-free survival; TNBC, Triple negative breast cancer; Vel, Veliparib.

**Figure 2:**
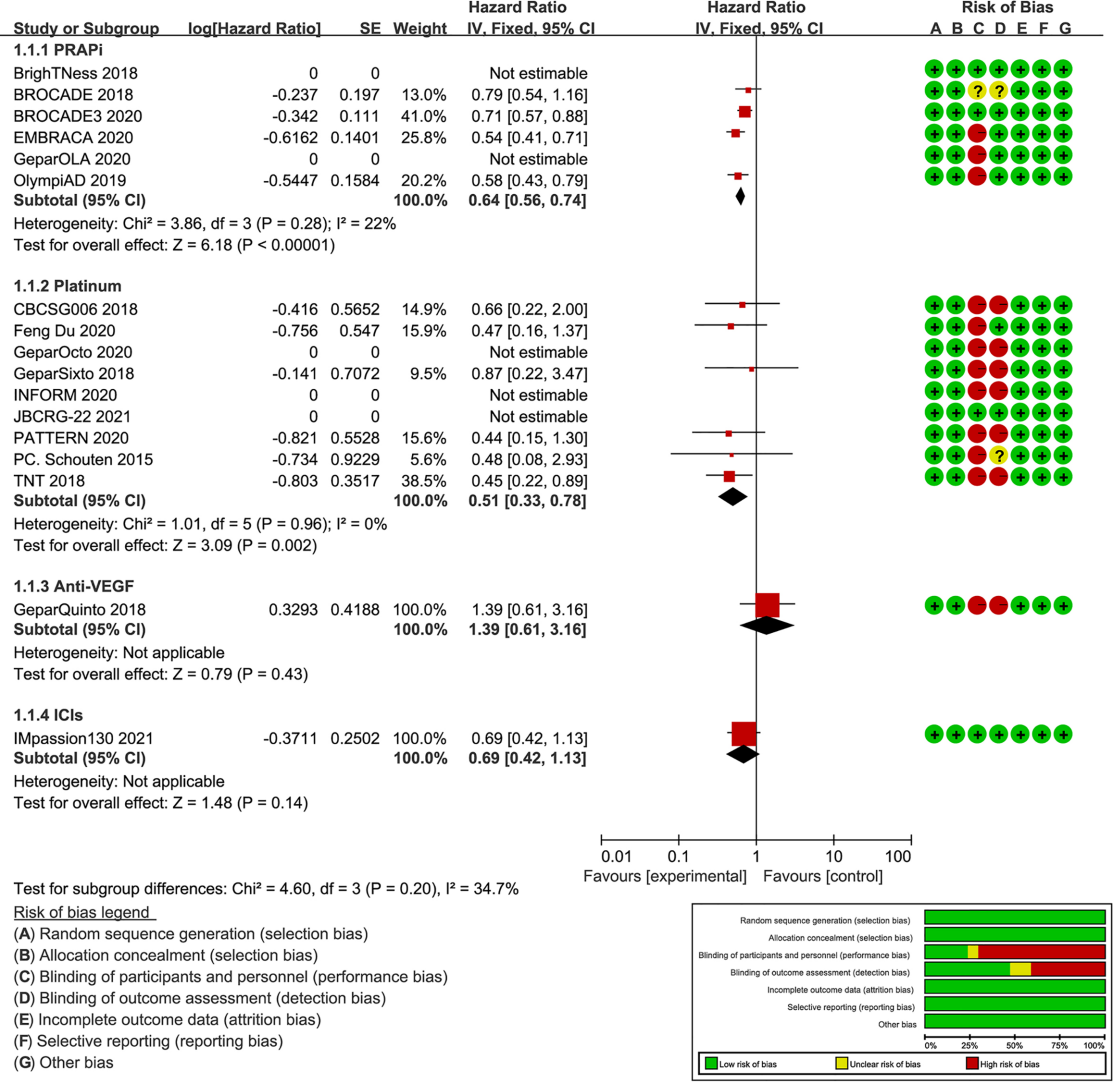
Forest plot of PFS results and risk of bias graph for each included study. On the left, the authors’ name, publication years, and the logHR results of individual studies are listed. Subgroup analysis is conducted according to different intervention drugs, including PARPi, platinum, anti-VEGF, and ICIs. The forest plot in the middle shows the PFS results of each study, and the center of diamonds represent the pooled HR. The center is left to vertical line, favoring the experimental groups. The horizontal tips represent the 95%CIs. The right side of the figure shows the risk of bias assessment for each study according to the Cochrane tool.

A total of 2,350 BC patients with gBRCA mutations were included. With a publication time cutoff from 2015 to 2021, all publications were published in the past 6 years. This is mainly because early-phase studies did not consider the presence of gBRCA mutations in patients. Four studies reported BC patients with gBRCA mutations as a subgroup (27, 35, 36, 38), and others considered BC patients with gBRCA mutations overall. The targeted agents analyzed included atezolizumab (Ate), talazoparib, olaparib, veliparib (Vel), bevacizumab (Beva), and platinum agents, including carboplatin and cisplatin (**Supplementary Table 1**). The subgroup meta-analysis of PFS results showed that PARPi (HR: 0.64; 95% CI: 0.56, 0.75; p<0.001) and platinum (HR: 0.51; 95% CI: 0.33, 0.78; p=0.002) were significantly better than the control (**Figure 2**). There was a study not included in the quantitative synthesis (26) because both the intervention and control were platinum agents + chemo according to our regimen classification method. Its pCR and ORR results showed no significant difference between paclitaxel plus carboplatin and eribulin plus carboplatin regimens for BRCA mutation TNBC patients (26). The included studies were all RCTs, and most of the studies have been publicly registered. Although there were deficiencies in the blinding method design, the overall design quality was good (**Figure 2**).

**Figure 3:**
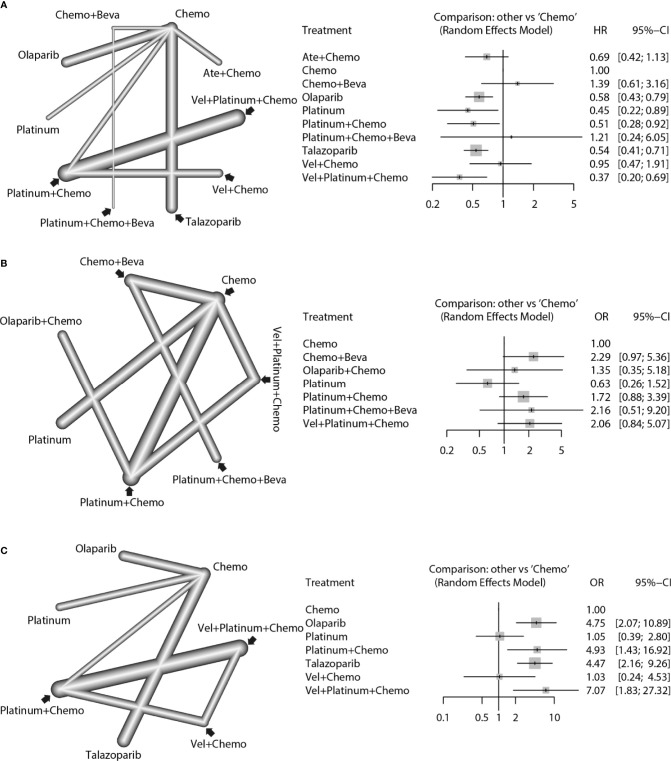
Network comparisons of PFS **(A)**, pCR **(B)**, and ORR **(C)** results for the strategies included in the analyses. The left side of the figure shows the network diagrams. Each dot indicates an intervention, and the edge between the two dots indicates that there is a direct comparison from the RCT. The thickness of the edge represents the precision of the comparisons. On the right, forest plots show the network meta-analysis results for all comparisons between Chemo and other interventions.

**Figure 4:**
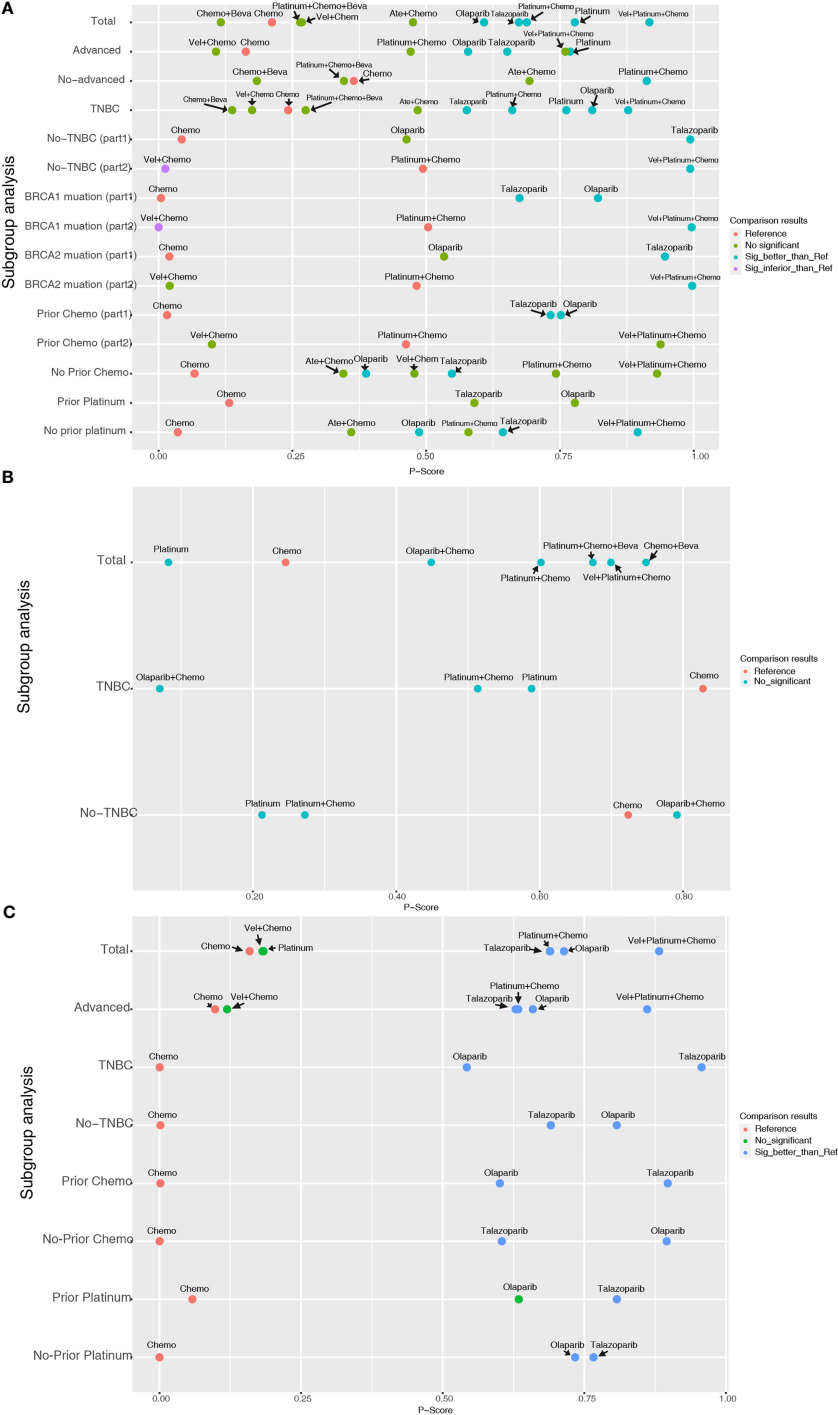
Entire and subgroup analyses of PFS **(A)**, pCR **(B)**, and ORR **(C)** results by network meta-analysis according to the P-score. Each dot represents an intervention, its ordinate represents the subgroup to which it belonged, and its abscissa represents the p-score results from the network meta-analysis. The various colors of the dots indicate whether there is a statistical difference compared with the reference (commonly Chemo).

**Figure 5:**
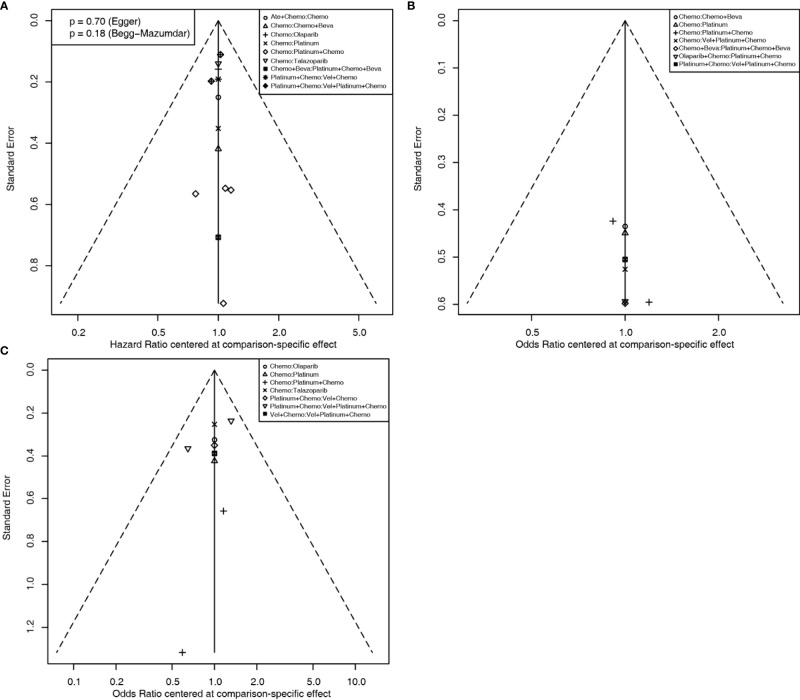
Comparison-adjusted funnel plots for assessing PFS **(A)**, pCR **(B)**, and ORR **(C)** results. Potential publication bias is assessed by whether the data point distribution is symmetrical, and the Egger’s and Begg’s test results are calculated if the number of data point is more than ten.

For the PFS analysis, ten regimens were included (**Figure 3A**). In the pairwise comparisons based on the network random effects model, olaparib (HR: 0.58; 95% CI: 0.43 – 0.79), platinum agents (HR: 0.45; 95% CI: 0.22 – 0.89), and talazoparib (HR: 0.54; 95% CI: 0.41 – 0.71) were significantly better than platinum-free chemotherapy (Chemo). The quality of evidence supporting the superiority of olaparib and talazoparib compared to Chemo was high (**Table 2**). In the results based on indirect comparisons, Vel + platinum agents + Chemo was also significantly better than Chemo (HR: 0.37; 95% CI: 0.20 – 0.69), with the highest p-score ranking. In the subgroup analysis, olaparib, talazoparib, and platinum agents were ideal regimens for advanced BC patients. The rank results were similar between the TNBC subgroup and the overall population. The non-TNBC patient population was divided into two network parts due to major differences. Talazoparib and Vel+platinum agent+Chemo remained ideal regimens. In non-TNBC patients and patients with BRCA2 mutations, talazoparib may have a relative advantage over olaparib. Olaparib and talazoparib were superior to Chemo regardless of whether the patient received prior chemotherapy. In addition, for patients who received prior platinum therapy, talazoparib and olaparib were not significantly better or worse than Chemo (**Figure 4A**). The study bias analysis did not show any publication bias (**Figure 5A**).

**Table 2:** The results of therapeutic regimens according to their relative effect and reliability.

Outcomes	Comparisons	No. of study	Direct Comparisons		Indirect Comparisons		Random Network Comparisons	
			HR/OR (95%CIs)	Quality	HR/OR (95%CIs)	Quality	HR/OR (95%CIs)	Quality
PFS	Ate+Chemo *vs.*							
	Chemo	1	0.69 (0.42 - 1.13)	Moderate†			0.69 (0.42 - 1.13)	Moderate
	Chemo+Beva				0.50 (0.19 - 1.29)	Low*†	0.50 (0.19 - 1.29)	Low
	Olaparib				1.19 (0.67 - 2.13)	Low‡	1.19 (0.67 - 2.13)	Low
	Platinum				1.54 (0.66 - 3.59)	Very low*‡	1.54 (0.66 - 3.59)	Very low
	Platinum+Chemo				1.36 (0.63 - 2.93)	Very low*‡	1.36 (0.63 - 2.93)	Very low
	Platinum+Chemo+Beva				0.57 (0.11 - 3.08)	Very low*‡	0.57 (0.11 - 3.08)	Very low
	Talazoparib				1.28 (0.73 - 2.24)	Low‡	1.28 (0.73 - 2.24)	Low
	Vel+Chemo				0.73 (0.31 - 1.72)	Very low*‡	0.73 (0.31 - 1.72)	Very low
	Vel+Platinum+Chemo				1.86 (0.84 - 4.11)	Low*†	1.86 (0.84 - 4.11)	Low
	Chemo *vs.*							
	Beva+Chemo	1	0.72 (0.32 - 1.63)	Very low*‡			0.72 (0.32 - 1.63)	Very low
	Olaparib	1	**1.72 (1.26 - 2.35)**	High			**1.72 (1.26 - 2.35)**	High
	Platinum	1	**2.23 (1.12 - 4.45)**	Moderate*			**2.23 (1.12 - 4.45)**	Moderate
	Platinum+Chemo	5	**1.97 (1.09 - 3.56)**	Moderate*			**1.97 (1.09 - 3.56)**	Moderate
	Platinum+Chemo+Beva				0.83 (0.17 - 4.15)	Very low*‡	0.83 (0.17 - 4.15)	Very low
	Talazoparib	1	**1.85 (1.41 - 2.44)**	High			**1.85 (1.41 - 2.44)**	High
	Vel+Chemo				1.06 (0.52 – 2.13)	Very low*‡	1.06 (0.52 – 2.13)	Very low
	Vel+Platinum+Chemo				**2.70 (1.45 – 5.03)**	Moderate*	**2.70 (1.45 – 5.03)**	Moderate
	Chemo+Beva *vs.*							
	Olaparib				**2.40 (1.00 - 5.76)**	Moderate*	**2.40 (1.00 - 5.76)**	Moderate
	Platinum				**3.10 (1.06 - 9.06)**	Moderate*	**3.10 (1.06 - 9.06)**	Moderate
	Platinum+Chemo				2.73 (0.99 – 7.52)	Low*†	2.73 (0.99 – 7.52)	Low
	Platinum+Chemo+Beva	1	1.15 (0.29 - 4.60)	Very low*‡			1.15 (0.29 - 4.60)	Very low
	Talazoparib				**2.57 (1.08 - 6.12)**	Moderate*	**2.57 (1.08 - 6.12)**	Moderate
	Vel+Chemo				1.47 (0.50 – 4.33)	Very low*‡	1.47 (0.50 – 4.33)	Very low
	Vel+Platinum+Chemo				**3.75 (1.34 – 10.51)**	Moderate*	**3.75 (1.34 – 10.51)**	Moderate
	Olaparib *vs.*							
	Platinum				1.29 (0.61 - 2.76)	Very low*‡	1.29 (0.61 - 2.76)	Very low
	Platinum+Chemo				1.14 (0.58 – 2.23)	Very low*‡	1.14 (0.58 – 2.23)	Very low
	Platinum+Chemo+Beva				0.48 (0.09 - 2.48)	Very low*‡	0.48 (0.09 - 2.48)	Very low
	Talazoparib				1.07 (0.71 - 1.63)	Low‡	1.07 (0.71 - 1.63)	Low
	Vel+Chemo				0.61 (0.28 - 1.32)	Low*†	0.61 (0.28 - 1.32)	Low
	Vel+Platinum+Chemo				1.57 (0.78 – 3.14)	Very low*‡	1.57 (0.78 – 3.14)	Very low
	Platinum *vs.*							
	Platinum+Chemo				0.88 (0.35 - 2.19)	Very low*‡	0.88 (0.35 - 2.19)	Very low
	Platinum+Chemo+Beva				0.37 (0.06 - 2.14)	Very low*‡	0.37 (0.06 - 2.14)	Very low
	Talazoparib				0.83 (0.40 - 1.74)	Very low*‡	0.83 (0.40 - 1.74)	Very low
	Vel+Chemo				0.47 (0.18 - 1.27)	Very low*‡	0.47 (0.18 - 1.27)	Very low
	Vel+Platinum+Chemo				1.21 (0.48 – 3.06)	Very low*‡	1.21 (0.48 – 3.06)	Very low
	Platinum+Chemo *vs.*							
	Platinum+Chemo+Beva				0.42 (0.08 - 2.35)	Very low*‡	0.42 (0.08 - 2.35)	Very low
	Talazoparib				0.94 (0.49 - 1.81)	Low‡	0.94 (0.49 - 1.81)	Low
	Vel+Chemo	1	0.54 (0.37 - 0.78)	Moderate*			0.54 (0.37 - 0.78)	Moderate
	Vel+Platinum+Chemo	2	**1.37 (1.14 - 1.66)**	High			**1.37 (1.14 - 1.66)**	High
	Platinum+Chemo+Beva *vs.*							
	Talazoparib				2.24 (0.44 - 11.46)	Very low*‡	2.24 (0.44 - 11.46)	Very low
	Vel+Chemo				1.28 (0.22 - 7.40)	Very low*‡	1.28 (0.22 - 7.40)	Very low
	Vel+Platinum+Chemo				3.26 (0.58 - 18.33)	Very low*‡	3.26 (0.58 - 18.33)	Very low
	Talazoparib *vs.*							
	Vel+Chemo				0.57 (0.27 - 1.21)	Low*†	0.57 (0.27 - 1.21)	Low
	Vel+Platinum+Chemo				1.46 (0.74 - 2.88)	Very low*‡	1.46 (0.74 - 2.88)	Very low
	Vel+Chemo *vs.*							
	Vel+Platinum+Chemo				**2.55 (1.68 - 3.88)**	Moderate*	**2.55 (1.68 - 3.88)**	Moderate
OS (part1)	Ate+Chemo *vs.*							
	Chemo	1	0.71 (0.39 - 1.29)	Low‡			0.71 (0.39 - 1.29)	Low
	Olaparib				0.79 (0.40 - 1.55)	Low‡	0.79 (0.40 - 1.55)	Low
	Platinum				0.74 (0.23 - 2.35)	Low‡	0.74 (0.23 - 2.35)	Low
	Talazoparib				0.84 (0.44 - 1.59)	Low‡	0.84 (0.44 - 1.59)	Low
	Chemo *vs.*							
	Olaparib	1	1.11 (0.81 - 1.52)	Moderate†			1.11 (0.81 - 1.52)	Moderate
	Platinum	1	1.04 (0.39 - 2.81)	Very low*‡			1.04 (0.39 - 2.81)	Very low
	Talazoparib	1	1.18 (0.93 - 1.49)	Moderate†			1.18 (0.93 - 1.49)	Moderate
	Olaparib *vs.*							
	Platinum				0.94 (0.33 - 2.65)	Very low*‡	0.94 (0.33 - 2.65)	Very low
	Talazoparib				1.06 (0.72 - 1.57)	Low‡	1.06 (0.72 - 1.57)	Low
	Platinum *vs.*							
	Talazoparib				1.13 (0.41 – 3.12)	Very low*‡	1.13 (0.41 – 3.12)	Very low
OS (part2)	Platinum+Chemo *vs.*							
	Vel+Chemo	1	0.67 (0.47 - 0.97)	Moderate*			**0.67 (0.47 - 0.97)**	Moderate
	Vel+Platinum+Chemo	2	1.13 (0.91 - 1.41)	Low*†			1.13 (0.91 - 1.41)	Low
	Vel+Chemo *vs.*							
	Vel+Platinum+Chemo				**1.68 (1.10 – 2.56)**	Moderate*	**1.68 (1.10 – 2.56)**	Moderate
pCR	Chemo *vs.*							
	Chemo+Beva	1	0.44 (0.19 – 1.03)	Low*†			0.44 (0.19 – 1.03)	Low
	Olaparib+Chemo				0.74 (0.19 - 2.85)	Very low*‡	0.74 (0.19 - 2.85)	Very low
	Platinum		1.59 (0.66 - 3.84)	Very low*‡			1.59 (0.66 - 3.84)	Very low
	Platinum+Chemo	1	0.58 (0.29 - 1.14)	Very low*‡			0.58 (0.29 - 1.14)	Very low
	Platinum+Chemo+Beva				0.46 (0.11 - 1.97)	Very low*‡	0.46 (0.11 - 1.97)	Very low
	Vel+Platinum+Chemo		0.53 (0.19 - 1.49)	Low‡	0.34 (0.06 - 2.27)	Very low*‡	0.48 (0.20 – 1.19)	Low
	Chemo+Beva *vs.*							
	Olaparib+Chemo				1.70 (0.34 - 8.35)	Very low*‡	1.70 (0.34 - 8.35)	Very low
	Platinum				**3.64 (1.07 - 12.39)**	Moderate *	3.64 (1.07 - 12.39)	Moderate
	Platinum+Chemo				1.33 (0.45 - 3.94)	Very low*‡	1.33 (0.45 - 3.94)	Very low
	Platinum+Chemo+Beva	1	1.06 (0.33 – 3.42)	Low*†			1.06 (0.33 - 3.42)	Low
	Vel+Platinum+Chemo				1.11 (0.32 - 3.83)	Very low*‡	1.11 (0.32 - 3.83)	Very low
	Olaparib+Chemo *vs.*							
	Platinum				2.14 (0.43 - 10.71)	Very low*‡	2.14 (0.43 - 10.71)	Very low
	Platinum+Chemo	1	0.78 (0.24 - 2.50)	Low‡			0.78 (0.24 - 2.50)	Low
	Platinum+Chemo+Beva				0.62 (0.09 - 4.51)	Very low*‡	0.62 (0.09 - 4.51)	Very low
	Vel+Platinum+Chemo				0.65 (0.15 - 2.82)	Low‡	0.65 (0.15 - 2.82)	Low
	Platinum *vs.*							
	Platinum+Chemo				0.36 (0.12 - 1.11)	Low*†	0.36 (0.12 - 1.11)	Low
	Platinum+Chemo+Beva				0.29 (0.05 - 1.59)	Very low*‡	0.29 (0.05 - 1.59)	Very low
	Vel+Platinum+Chemo				0.30 (0.09 - 1.07)	Low*†	0.30 (0.09 - 1.07)	Low
	Platinum+Chemo *vs.*							
	Platinum+Chemo+Beva				0.80 (0.16 - 3.96)	Very low*‡	0.80 (0.16 - 3.96)	Very low
	Vel+Platinum+Chemo	1	0.77 (0.29 - 2.07)	Low‡	1.16 (0.16 – 8.20)	Very low*‡	0.84 (0.35 - 2.02)	Low
	Platinum+Chemo+Beva *vs.*							
	Vel+Platinum+Chemo	1	1.05 (0.19 - 5.76)	Very low*‡			1.05 (0.19 - 5.76)	Very low
ORR	Chemo *vs.*							
	Olaparib	1	**0.21 (0.09 - 0.48)**	High			**0.21 (0.09 - 0.48)**	High
	Platinum	1	0.96 (0.36 - 2.56)	Very low*‡			0.96 (0.36 - 2.56)	Very low
	Platinum+Chemo	2	**0.20 (0.06 - 0.70)**	Moderate*			**0.20 (0.06 - 0.70)**	Moderate
	Talazoparib	1	**0.22 (0.11 - 0.46)**	High			**0.22 (0.11 - 0.46)**	High
	Vel+Chemo				0.97 (0.22 - 4.25)	Very low*‡	0.97 (0.22 - 4.25)	Very low
	Vel+Platinum+Chemo				0.14 (0.04 - 0.55)	Moderate*	0.14 (0.04 - 0.55)	Moderate
	Olaparib *vs.*							
	Platinum				**4.54 (1.25 - 16.46)**	Moderate*	**4.54 (1.25 - 16.46)**	Moderate
	Platinum+Chemo				0.96 (0.22 - 4.27)	Very low*‡	0.96 (0.22 - 4.27)	Very low
	Talazoparib				1.06 (0.35 - 3.20)	Low‡	1.06 (0.35 - 3.20)	Low
	Vel+Chemo				4.60 (0.84 - 25.07)	Low*†	4.60 (0.84 - 25.07)	Low
	Vel+Platinum+Chemo				0.67 (0.14 - 3.29)	Very low*‡	0.67 (0.14 - 3.29)	Very low
	Platinum *vs.*							
	Platinum+Chemo				0.21 (0.04 - 1.03)	Low*†	0.21 (0.04 - 1.03)	Low
	Talazoparib				0.23 (0.07 - 0.80)	Moderate*	0.23 (0.07 - 0.80)	Moderate
	Vel+Chemo				1.01 (0.17 - 5.99)	Very low*‡	1.01 (0.17 - 5.99)	Very low
	Vel+Platinum+Chemo				0.15 (0.03 - 0.79)	Moderate*	0.15 (0.03 - 0.79)	Moderate
	Platinum+Chemo *vs.*							
	Talazoparib				1.10 (0.26 - 4.61)	Very low*‡	1.10 (0.26 - 4.61)	Very low
	Vel+Chemo	1	**3.95 (1.66 - 9.43)**	Moderate*	**18.6 (1.79 - 192.75)**	Moderate*	**4.77 (2.11 - 10.77)**	Moderate
	Vel+Platinum+Chemo				0.70 (0.40 - 1.21)	Low*†	0.70 (0.40 - 1.21)	Low
	Talazoparib *vs.*							
	Vel+Chemo				4.33 (0.83 - 22.51)	Low*†	4.33 (0.83 - 22.51)	Low
	Vel+Platinum+Chemo				0.63 (0.14 - 2.94)	Very low*‡	0.63 (0.14 - 2.94)	Very low
	Vel+Chemo *vs.*							
	Vel+Platinum+Chemo	1	**0.11 (0.05 - 0.29)**	Moderate*	0.44 (0.06 - 3.15)	Very low*‡	0.15 (0.06 - 0.34)	Low
SAE (part1)	Chemo *vs.*							
	Olaparib	1	1.59 (0.97 - 2.62)	Moderate†			1.59 (0.97 - 2.62)	Moderate
	Platinum	2	0.89 (0.43 - 1.83)	Very low*‡			0.89 (0.43 - 1.83)	Very low
	Talazoparib	1	0.94 (0.58 - 1.50)	Low‡			0.94 (0.58 - 1.50)	Low
	Olaparib *vs.*							
	Platinum				0.56 (0.23 - 1.34)	Very low*‡	0.56 (0.23 - 1.34)	Very low
	Talazoparib				0.59 (0.30 - 1.16)	Moderate†	0.59 (0.30 - 1.16)	Moderate
	Platinum *vs.*							
	Talazoparib				1.05 (0.44 - 2.50)	Very low*‡	1.05 (0.44 - 2.50)	Very low
SAE (part2)	Platinum+Chemo *vs.*							
	Vel+Chemo	1	1.94 (0.96 - 3.92)	Low*†	0.70 (0.11 - 4.55)	Very low*‡	1.71 (0.89 - 3.30)	Low
	Vel+Platinum+Chemo	2	1.09 (0.61 - 1.92)	Very low*‡			1.09 (0.61 - 1.92)	Very low
	Vel+Chemo *vs.*							
	Vel+Platinum+Chemo	1	0.71 (0.36 - 1.38)	Very low*‡	0.24 (0.03 - 1.82)	Very low*‡	0.63 (0.34 - 1.20)	Very low

CIs, confidence intervals; pCR, pathologic complete response; HR, hazard ratio; OR, odds ratio; ORR objective response rate; OS, overall survival; SAEs, serious adverse effects; PFS, Progression-free survival.

Bold means significant difference (p < 0.05).

*Study design limitation; †Imprecise; ‡Very imprecise; :inconsistency.

In the OS results, due to the large differences between subgroups, the analysis of regimens was divided into two parts (**Supplementary Figures 1A, B**). The first analysis included the Ate+Chemo, Chemo, olaparib, platinum agent, and talazoparib regimens, but no significant difference was identified by pairwise comparisons. The second analysis included platinum agent+Chemo, Vel+Chemo, and Vel+platinum agent+Chemo. Among them, Vel+Chemo was inferior to platinum agent+Chemo (HR: 1.48; 95% CI: 1.03 - 2.13), and Vel+platinum agent+Chemo was significantly better than Vel+Chemo (HR: 1.68; 95% CI: 1.10-2.56) (**Table 2**). In the subgroup analysis, olaparib was found to be significantly better than Chemo only in the population who did not receive prior chemotherapy (**Supplementary Figure 2A**).

In the pCR analysis, only Chemo+Beva had a significant advantage (OR: 3.64; 95% CI: 1.07-12.39) over the platinum agent (**Figure 3B** and **Table 2**). The application of platinum agents alone was not ideal for the improvement of pCR. In addition, the small sample size affected the credibility of the subgroup results (**Figure 4B**). The study bias analysis did not show any publication bias (**Figure 5B**).

In the ORR analysis, Chemo was significantly inferior to olaparib (OR: 4.75; 95% CI: 2.07 - 10.89), platinum agent+Chemo (OR: 4.93; 95% CI: 1.43-16.92), and talazoparib (OR: 4.47; 95% CI: 2.16 – 9.26) (**Figure 3C**). Olaparib was significantly better than platinum agents (OR: 4.54; 95% CI: 1.25 - 16.46). Platinum agent+Chemo was also significantly better than Vel+Chemo (OR: 4.77; 95% CI: 2.11 - 10.77) (**Table 2**). In the subgroup analysis, the rank results of advanced patients were similar to those of the overall patients. Olaparib and talazoparib both showed significant advantages over Chemo, except for in patients who received prior platinum therapy (**Figure 4C**). The study bias analysis did not show any publication bias (**Figure 5C**). In the SAE analysis, there were no significant differences in SAEs between the two subgroups (**Supplementary Figures 1C, D** and **Supplementary Figure 2B**).

**Figure 6:**
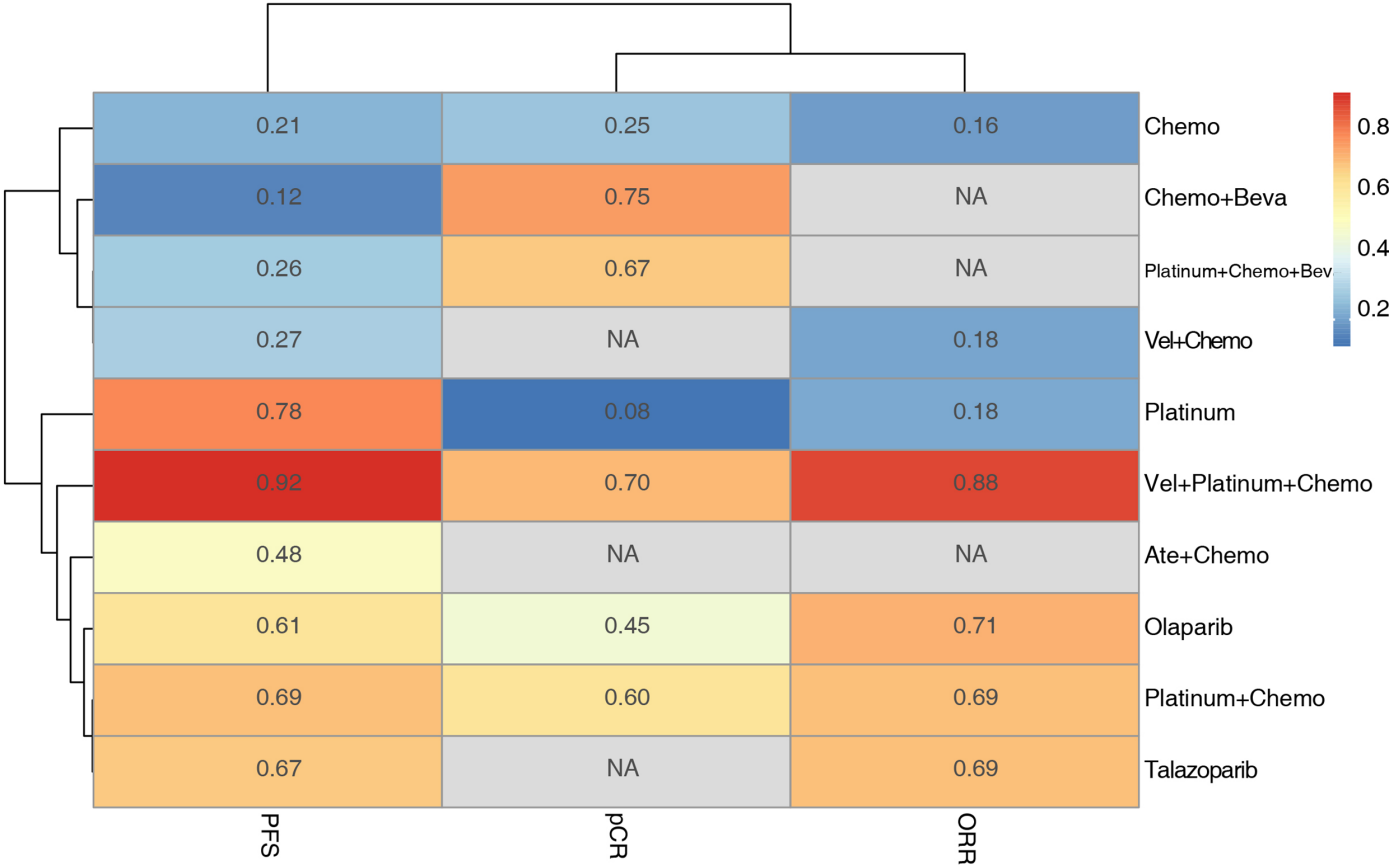
Hierarchical clustering of PFS pCR and ORR results based on the P-score. The numbers and colors in the grid represent the P-score value of each intervention method for each outcome, and the lines on the left and upper side represent the similarities among the elements by K-means cluster analysis.

K-means cluster analysis was performed on the P-score results of PFS, pCR, and ORR. The results showed that Vel + platinum agents + Chemo exhibited high effectiveness. Ate+ Chemo, olaparib, talazoparib, platinum agent + chemo, and platinum agent alone had moderate effectiveness. Others had relative low effectiveness (**Figure 6**). However, there were still missing values in the cluster analysis. More research is needed to correct the cluster results.

## Discussion

In this study, the effect and safety of therapeutic regimens in BC patients with BRCA mutations were analyzed by network meta-analysis. The PFS analysis suggested that olaparib, talazoparib, and Vel+platinum agent+Chemo were ideal regimens for overall, TNBC, and advanced BC patients with gBRCA mutations. It was also shown that talazoparib and olaparib had no obvious advantages over Chemo in patients who received prior platinum treatment. The OS analysis showed that olaparib was significantly better than Chemo only in the population who did not receive prior chemotherapy. In the pCR analysis, only Chemo+Beva was significantly better than the platinum agent. In the ORR analysis, olaparib and talazoparib had significant advantages over Chemo. However, olaparib had a relatively weak effect on patients who received prior platinum treatment. In addition, there were no significant differences in SAEs.

Mutations in the tumor suppressor gene BRCA1/2 cause HRR dysfunction, and in tumors with such mutations, PARP inhibition more effectively kills tumor cells, resulting in synthetic lethality (42). PARP1, which is mainly involved in single-strand DNA break repair, is the main target of PARPis. For tumor cells without HRR dysfunction, DNA damage caused by these agents will be repaired through HRR pathways. However, when HRR dysfunction occurs, such as HRR dysfunction caused by gBRCA mutation, DNA damage caused by PARPis can produce effective cytotoxicity and induce apoptosis. The results of this work prove that olaparib and talazoparib can prolong the PFS of patients with BRCA-mutated BC, and these agents are also suitable for TNBC and advanced BC patients. PARPis did not increase the SAE risk, suggesting that they specifically affected cancer cells and not normal cells. However, in the OS analysis, olaparib was beneficial compared with Chemo only in the TNBC subgroup. No other regimens were found to provide survival benefits to BC patients with gBRCA mutations.

Vel is a selective PARP1/2 inhibitor with relatively weak affinity, while olaparib and talazoparib have relatively strong affinity. These agents cannot cause the same scale of synthetic lethality (43). However, Vel mainly selectively inhibits the activity of PARP without holding the PARP protein to DNA damage repair intermediates, and combination regimens of Vel and platinum agents have been tested (44). PARP-1 has also been suggested to be a marker of the response to DNA-damaging platinum agents, which also supports the combination of Vel and platinum (45). This study showed the significant therapeutic advantage of Vel+platinum agent+Chemo but also showed that for non-TNBC patients, patients with BRCA1 mutations, and patients who do not receive prior chemotherapy, Vel+platinum agent will even be significantly inferior to platinum agent+Chemo. In patients who received prior platinum therapy, the Vel+Chemo regimen achieved significantly inferior OS compared to the platinum agent+Chemo regimen.

There is evidence that patients with gBRCA mutations show overexpression of vascular endothelial growth factor, angiopoietin-1, and angiopoietin-2 (46, 47). This finding supports the application of Beva to inhibit angiogenesis and cause hypoxia-related DNA damage and synthetic lethal reactions with few adverse events. Our analysis showed that Beva can significantly improve the pCR rate, but in the P-score ranking results of PFS, Beva-Chemo was even inferior to Chemo alone, although the difference was not statistically significant. The pCR and PFS were also different. In general, Beva combined with Chemo should not be recommended based on the survival outcomes.

However, whether PARPis are suitable for patients with gBRCA mutations who have received prior platinum agents still needs to be clarified. In the PFS subgroup analysis, olaparib and talazoparib were not significantly superior to Chemo in patients who had received prior platinum agents. The abovementioned differences between pCR and PFS and the poor effect of PARPis in patients who received prior platinum agents indicate that patients with gBRCA mutations who have received prior platinum therapy can regain BRCA function *via* additional mutations or the activation of other HRR mechanisms (48). Cross-resistance between platinum agents and PARPis has also been suggested. Therefore, the combined application of PARPis and platinum drugs can be used to maximize the therapeutic benefits in patients with gBRCA mutations. However, based on concerns about SAEs, especially hematological toxicity, only the combination of veliparib and platinum-related chemotherapy has been tested in RCTs, and this combination showed benefits that were similar to those of olaparib and talazoparib in patients with gBRCA mutations. However, for patients who received prior chemotherapy, Vel+platinum agent+Chemo also did not achieve significant PFS benefits compared to platinum agent+Chemo. Therefore, for this population, olaparib and talazoparib are still the best strategies.

One study (MEDIOLA) was excluded because it used a non-RCT design, and this study showed that the combination of olaparib and durvalumab has promising antitumor activity and safety that are similar to those of olaparib and durvalumab monotherapies (49). There are also some important ongoing RCTs. In one study, the PARPi niraparib showed superior survival results compared to the physician’s choice Chemo, but the final results were awaited (NCT01905592). In another study, the novel PARPi fluzoparib was combined with apatinib for the treatment of patients with gBRCA-mutated HER2-negative metastatic BC (NCT04296370). Olaparib combined with the PD-1 inhibitor pembrolizumab (NCT04191135), the PD-L1 antibody atezolizumab (NCT02849496), or durvalumab (NCT03167619) is being tested for the treatment of patients with gBRCA1/2-mutated BC. In addition, olaparib combined with ceralasertib or adavosertib is being used for TNBC with the gBRCA mutation (NCT03330847). With the completion of these RCTs, more therapeutic regimens and more accurate results will be provided to help uncover the ideal regimens for patients with gBRCA-mutated BC.

There are still several limitations in this work. First, due to the differences in groups, some outcomes and subgroup results were divided into two parts. Second, this work only analyzed patients with gBRCA mutations; patients with BRCA epigenetic modifications were not analyzed because epigenetic modification and expression levels might change with disease development and treatment. Patients who have loss of BRCA function due to causes besides mutation have more sensitivity to platinum therapy than those who have gBRCA mutation (50). Third, a variety of cytotoxic agents in platinum-free Chemo regimens (anthracyclines and alkylating agents) still have DNA-damaging effects. This diversity of cytotoxic agents in the Chemo arm increased the heterogeneity of the results, which was difficult to eliminate in this study.

## Data Availability Statement

The original contributions presented in the study are included in the article/**Supplementary Material**. Further inquiries can be directed to the corresponding author.

## Author Contributions

YJ and X-YM: Conceptualization, Methodology, Writing - Original draft preparation. CM: Methodology. N-ND and L-HL: Data curation, Software. ZH and X-YW: Software, Visualization. Z-YS: Software, Validation. R-JC: Supervision, Writing - Reviewing and Editing. YJ and X-YM contribute equally to this work. All authors contributed to the article and approved the submitted version.

## Funding

Youth Innovative Talents Training Program in General Undergraduate Colleges and Universities in Heilongjiang Province (No.2020-QC23).

## Conflict of Interest

The authors declare that the research was conducted in the absence of any commercial or financial relationships that could be construed as a potential conflict of interest.

## Publisher’s Note

All claims expressed in this article are solely those of the authors and do not necessarily represent those of their affiliated organizations, or those of the publisher, the editors and the reviewers. Any product that may be evaluated in this article, or claim that may be made by its manufacturer, is not guaranteed or endorsed by the publisher.

## Supplementary Material

Supplementary Figure 1 Network comparisons of OS **(A, B)** and SAE **(C, D)** results for the strategies included in the analyses. The left side of the figure shows the network diagrams; each dot indicates an intervention, and the edge between the two dots indicates that there is a direct comparison from the RCT. The thickness of the edge represents the precision of the comparisons. On the right side, forest plots show the network meta-analysis results for all comparisons between Chemo (or Platinum+Chemo) and other interventions.

Supplementary Figure 2 Entire and subgroup analyses of OS **(A)** and SAE **(B)** results by network meta-analysis according to the P-score. Each dot represents an intervention, its ordinate represents the subgroup to which it belonged, and its abscissa represents the p-score results from the network meta-analysis. The various colors of the dots indicate whether there is a statistical difference compared with the reference (commonly Chemo).
