# Supplementary figures and images for: Effect and Safety of Therapeutic Regimens for Patients With Germline BRCA Mutation-Associated Breast Cancer: A Network Meta-Analysis
Source: Front Oncol. 2021 Aug 20;11:718761. doi: 10.3389/fonc.2021.718761 (PMC8417748; doi:10.3389/fonc.2021.718761)

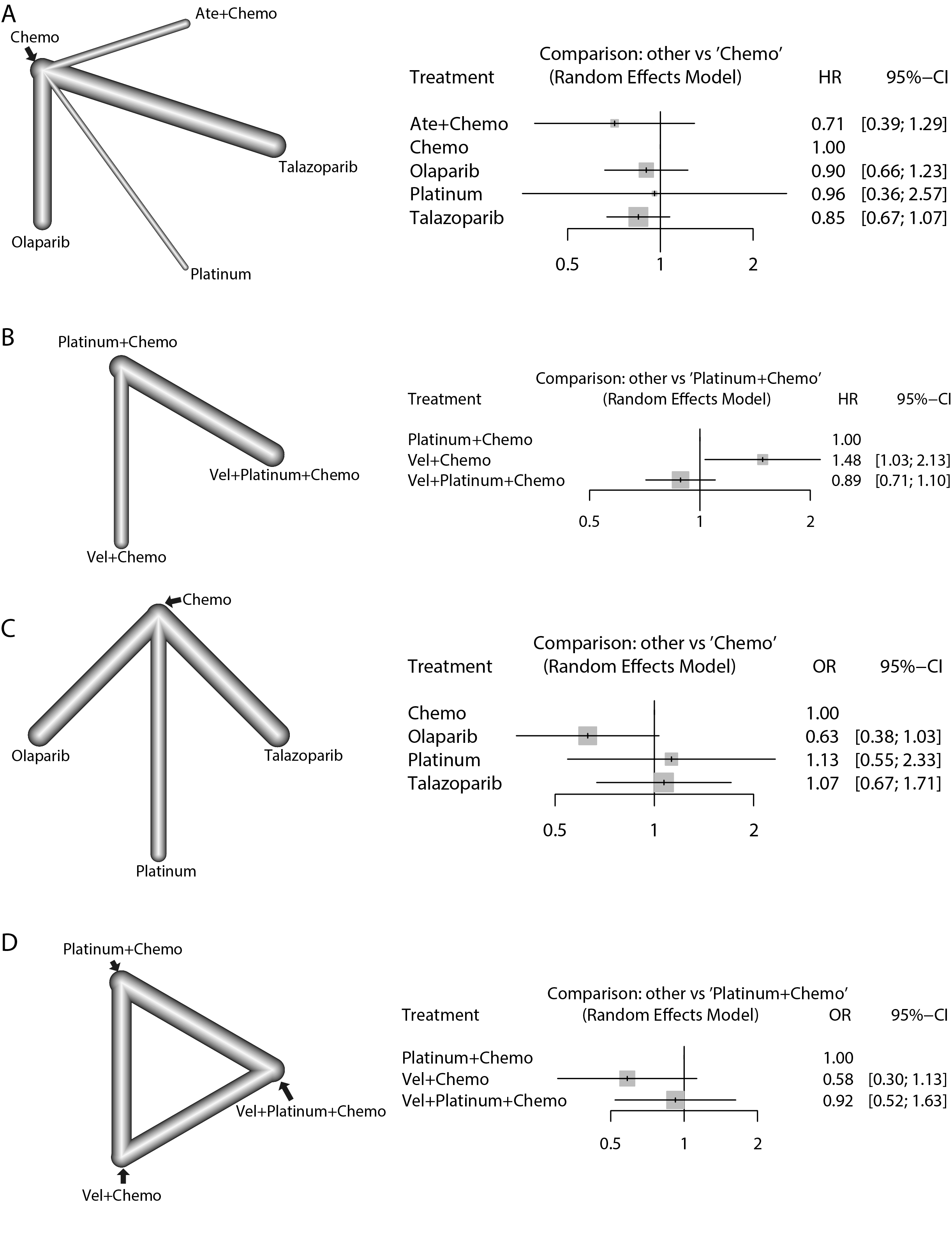

Supplement: Supplementary Figure 1 — Network comparisons of OS (A, B) and SAE (C, D) results for the strategies included in the analyses. The left side of the figure shows the network diagrams; each dot indicates an intervention, and the edge between the two dots indicates that there is a direct comparison from the RCT. The thickness of the edge represents the precision of the comparisons. On the right side, forest plots show the network meta-analysis results for all comparisons between Chemo (or Platinum+Chemo) and other interventions. [file Image_1.jpeg]

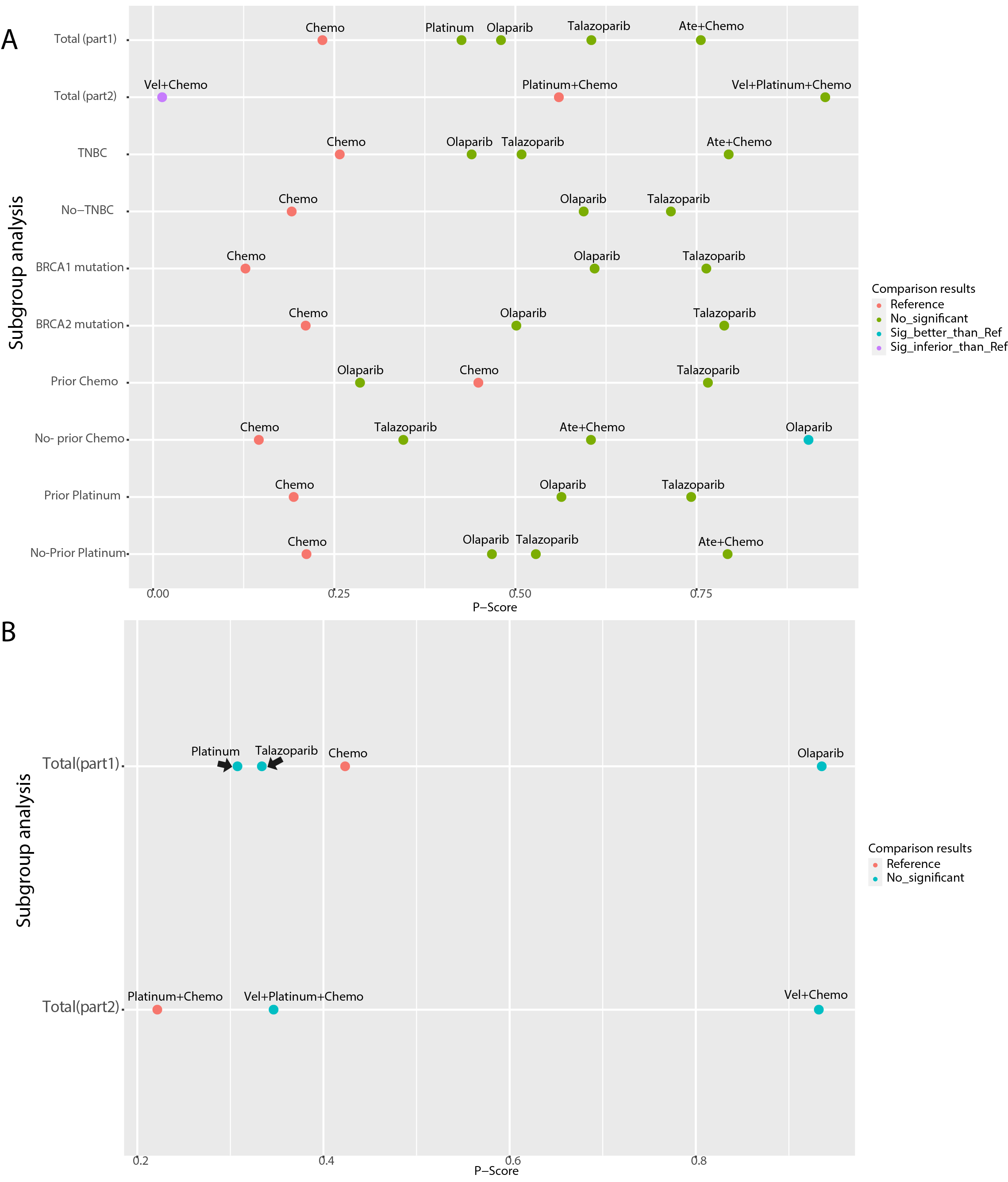

Supplement: Supplementary Figure 2 — Entire and subgroup analyses of OS (A) and SAE (B) results by network meta-analysis according to the P-score. Each dot represents an intervention, its ordinate represents the subgroup to which it belonged, and its abscissa represents the p-score results from the network meta-analysis. The various colors of the dots indicate whether there is a statistical difference compared with the reference (commonly Chemo). [file Image_2.jpeg]
