# Supplementary material for: Effect and Safety of Therapeutic Regimens for Patients With Germline BRCA Mutation-Associated Breast Cancer: A Network Meta-Analysis
Source: Front Oncol. 2021 Aug 20;11:718761. doi: 10.3389/fonc.2021.718761 (PMC8417748; doi:10.3389/fonc.2021.718761)
Supplement: Supplementary file 3 [file Table_1.docx]

STable 1. Details of interventions in each included studies

| **Study** | **Study Abbr.** | **Register ID** | **Interventions** | **Abbr.** | **Control** | **Abbr.** |
| --- | --- | --- | --- | --- | --- | --- |
| LA.Emens 2021 [25] | IMpassion130 | NCT02425891 | Atezolizumab; nab Paclitaxel | Ate+Chemo | Placebo; nab paclitaxel | Chemo |
| N. Masuda 2021 [26] | JBCRG-22 | UMIN000023162 | Paclitaxel; Carboplatin | Platinum+Chemo | Eribulin; Carboplatin | Platinum+Chemo |
| Ke-Da Yu 2020[27] | PATTERN | NCT01216111 | Paclitaxel;Carboplatin | Platinum+Chemo | Cyclophosphamide;epirubicin;fluorouracil;docetaxel | Chemo |
| Nadine Tung 2020[28] | INFORM | NCT01670500 | Doxorubicin;Cyclophosphamide | Chemo | Cisplatin | Platinum |
| Hope S. Rugo 2020[29] | EMBRACA | NCT01945775 | Talazoparib | Talazoparib | Physician's choice of chemotherapy (capecitabine, eribulin, gemcitabine, vinorelbine) | Chemo |
| Véronique Diéras 2020[30] | BROCADE3 | NCT02163694 | Veliparib; carboplatin; paclitaxel | Vel+Platinum+Chemo | Placebo; carboplatin; paclitaxel | Platinum+Chemo |
| Esther Pohl-Rescigno 2020[31] | GeparOcto | NCT02125344 | Epirubicin; paclitaxel;cyclophosphamide | Chemo | Carboplatin; paclitaxel; doxorubicin | Platinum+Chemo |
| PA. Fasching 2020[32] | GeparOLA | NCT02789332 | Paclitaxel;olaparib;epirubicin; cyclophosphamide | Olaparib+Chemo | Paclitaxel; carboplatinum; epirubicin; cyclophosphamide | Platinum+Chemo |
| Feng Du 2020[33] | NA | NA | Docetaxel/paclitaxel; carboplatin | Platinum+Chemo | Docetaxel/paclitaxel;epirubicin; cyclophosphamide | Chemo |
| ME Robson 2019[34] | OlympiAD | NCT02000622 | Olaparib | Olaparib | Physician's choice of chemotherapy(capecitabine, vinorelbine, or eribulin) | Chemo |
| J.Zhang 2018[35] | CBCSG006 | NCT01287624 | Cisplatin;gemcitabine | Platinum+Chemo | Paclitaxel;gemcitabine | Chemo |
| Andrew Tutt 2018[36] | TNT | NCT00532727 | Carboplatin | Platinum | Docetaxel | Chemo |
| S. Loibl 2018[37] | GeparSixto | NCT01426880 | Paclitaxel;doxorubicin;bevacizumab(TNBC) | Beva+Chemo | Paclitaxel;doxorubicin;carboplatin;bevacizumab(TNBC) | Beva+Platinum+Chemo |
| S. Loibl 2018[38] | BrighTNess | NCT02032277 | Paclitaxel;carboplatin;veliparib | Vel+Platinum+Chemo | Paclitaxel+carboplatin | Platinum+Chemo |
|  |  |  | Paclitaxel | Chemo |  |  |
| HS. Han 2018[39] | BROCADE | NCT01506609 | Veliparib;carboplatin;paclitaxel | Vel+Platinum+Chemo | Veliparib;temozolomide | Vel+Chemo |
|  |  |  | Placebo;carboplatin;paclitaxel | Platinum+Chemo |  |  |
| PA. Fasching 2018[40] | GeparQuinto | NCT00567554 | Bevacizumab;epirubicin;cyclophosphamide;docetaxel | Beva+Chemo | Epirubicin;cyclophosphamide;docetaxel | Chemo |
| PC. Schouten 2015[41] | NA | NA | Carboplatin;thiotepa;5-fluorouracil;epirubicin;cyclphosphamide | Platinum+Chemo | 5-fluorouracil+epirubicin+cyclphosphamide | Chemo |

Abbreviations: Ate: Atezolizumab; Beva: Bevacizumab; Chemo: Chemotherapy; NA: not available; Vel: Veliparib.
